# Gene expression profiles of mucosal biopsy specimens in children with eosinophilic gastritis

**DOI:** 10.1186/1939-4551-8-S1-A176

**Published:** 2015-04-08

**Authors:** Tetsuo Shoda, Ichiro Nomura, Akio Matsuda, Kyoko Futamura, Kanami Orihara, Hideaki Morita, Katsuhiro Arai, Hirotaka Shimizu, Masami Narita, Yukihiro Ohya, Yoshiyuki Yamada, Hirohisa Saito, Kenji Matsumoto

**Affiliations:** 1 National Research Institute for Child Health & Development, Japan; 2 Gunma Children's Medical Center, Japan

## Background

Eosinophilic gastrointestinal disorders (EGID) are clinicopathologically characterized by massive eosinophilic infiltration into the gastrointestinal tract and are classified into eosinophilic esophagitis (EoE), gastritis (EG), gastroenteritis, enteritis and colitis according to the site of infiltration. Studies of the pathogenic mechanism of EoE, whose incidence and prevalence are increasing in Western countries, revealed that eotaxin-3 plays a crucial role in inducing selective recruitment of eosinophils into the esophageal epithelium. In contrast, the pathogenic mechanism of EG remains obscure. In order to elucidate whether EG’s pathogenic mechanism is similar to that of EoE, we performed transcriptome analysis of gastric biopsy specimens from EG patients and compared the identified gene signature to the previous microarray data for EoE patients (J Clin Invest, 116:536-47, 2006).

## Methods

We enrolled pediatric EG patients (n = 5) and age-matched controls (n = 5) who, after we obtained informed consent from their guardians, underwent gastrointestinal endoscopy due to clinical symptoms. EG was diagnosed on the basis of ≥30 eosinophils/HPF, limited to the stomach, according to Lwin’s criteria (Modern Pathology 24:556-63, 2011). The gene expression profiles of the gastric biopsies were assessed using microarray technology with Agilent SurePrint G3 Human GE 8 x 60k. The differentially expressed genes of EG and EoE were compared by systematic analysis using the NextBio search engine.

## Results

Of 42,545 transcripts represented on the microarray, 2,282 were differentially expressed between the EG and control samples (≥2 fold change and adjusted p-value of <0.05). In agreement with a previous study on EoE patients, eotaxin-3 was the most upregulated (>2,000-fold) gene compared with the control subjects. Of the 2,282 transcripts composing the EG-related gene signature, 58, including eotaxin-3, were identified as commonly upregulated genes in EoE.

## Conclusions

Our results suggest that eotaxin-3 plays a crucial effector role in the pathogenesis of EG as well as EoE. On the other hand, 97.5% of the gene signature we identified for EG was distinct from that previously identified for EoE, suggesting that distinct mechanisms may be involved in the pathogenesis of EG and EoE.Background

